# Immediate Remote Ischemic Postconditioning Reduces Brain Nitrotyrosine Formation in a Piglet Asphyxia Model

**DOI:** 10.1155/2016/5763743

**Published:** 2016-06-09

**Authors:** Eridan Rocha-Ferreira, Brogan Rudge, Michael P. Hughes, Ahad A. Rahim, Mariya Hristova, Nicola J. Robertson

**Affiliations:** ^1^Preclinical Neonatal Neuroprotection Group, UCL EGA Institute for Women's Health, London WC1E 6BT, UK; ^2^Department of Pharmacology, UCL School of Pharmacy, London WC1N 1AX, UK; ^3^Perinatal Brain Group, UCL EGA Institute for Women's Health, London WC1E 6HX, UK

## Abstract

Remote ischemic postconditioning (RIPostC) is a promising therapeutic intervention that could be administered as an alternative to cooling in cases of perinatal hypoxia-ischemia (HI). In the current study we hypothesized that RIPostC in the piglet model of birth asphyxia confers protection by reducing nitrosative stress and subsequent nitrotyrosine formation, as well as having an effect on glial immunoreactivity. Postnatal day 1 (P1) piglets underwent HI brain injury and were randomised to HI (control) or HI + RIPostC. Immunohistochemistry assessment 48 hours after HI revealed a significant decrease in brain nitrotyrosine deposits in the RIPostC-treated group (*p* = 0.02). This was accompanied by a significant increase in eNOS expression (*p* < 0.0001) and decrease in iNOS (*p* = 0.010), with no alteration in nNOS activity. Interestingly, RIPostC treatment was associated with a significant increase in GFAP (*p* = 0.002) and IBA1 (*p* = 0.006), markers of astroglial and microglial activity, respectively. The current study demonstrates a beneficial effect of RIPostC therapy in the preclinical piglet model of neonatal asphyxia, which appears to be mediated by modulation of nitrosative stress, despite glial activation.

## 1. Introduction

Hypoxic-ischemic encephalopathy (HIE) occurs after intrapartum asphyxia [1] and is responsible for 23% of neonatal deaths worldwide [2]. Hypothermia therapy has been established as standard clinical care for infants diagnosed with moderate to severe neonatal encephalopathy (NE) in the developed world. Cooling started within six hours of birth ameliorates secondary energy failure and cell death, significantly lowering the risk of death and severe disability in treated infants [3, 4]. In the UK, 45% of infants have adverse outcome after HIE despite cooling, with 25% dying and 20% developing cognitive cerebral palsy and other life-long debilitating conditions [5]. Adjunct therapies for hypothermia are needed to enhance overall protection and improve outcome. In low and mid resource settings where cooling is not routine, alternative therapies may be important.

The developing neonatal brain is particularly vulnerable to oxidative stress because free-radical scavenging systems have not yet matured, resulting in insufficient synthesis of antioxidant enzymes/scavengers following injury [6]. The free-radical nitric oxide (NO) is an ubiquitous neurotransmitter and an important signalling molecule with multiple functions within the CNS. In the brain, NO is synthetized from arginine, nicotinamide adenine dinucleotide phosphate (NADPH), and oxygen by three distinct isoforms of NO synthase (NOS). Neuronal NOS (nNOS) is a calcium-dependent enzyme that is upregulated during ischemia. Resulting NO production reacts with ROS to produce several radicals detrimental to neuronal survival [7–12]. Inducible NOS (iNOS) is a calcium-independent enzyme associated with inflammatory markers and can be produced by macrophages and various other cells under pathological conditions. Like nNOS, iNOS has also shown a link with neuronal loss [6, 8, 9, 11, 13]. Calcium-dependent endothelial NOS (eNOS) enzyme, unlike nNOS and iNOS isoenzymes, is thought to be protective under ischemic conditions, potentially as a result of its vasodilating effect and subsequent improvement in cerebral perfusion [9, 11, 14–17]. Oxidative stress results in excessive production of NO within different brain regions. NO then combines with superoxide radicals to produce peroxynitrite [18, 19]. Nitrotyrosine is a product of tyrosine nitration from peroxynitrite, and its formation in proteins is an indicator of cell damage. This has been shown both in animal models as well as in clinical cases of brain perinatal asphyxia [11, 20–23]. In addition to directly causing apoptotic cell death in the brain, oxidative stress products induce a profound inflammatory response characterised by neuroglial activation [24]. Reactive oxygen species and important transcription factors such as the inflammatory and antiapoptotic NF-*κ*B [11] stimulate astrocytes and microglia, which respond by secreting a number of proinflammatory cytokines such as interleukin 6 (IL-6) and tumour necrosis factor alpha (TNF-*α*) [19, 25].

RIPostC is the application of “brief intermittent cycles of ischemia alternating with reperfusion” [26] in the limbs after an ischemic insult. RIPostC has been shown to reduce cerebral ischemia-induced infarct size and neuronal apoptosis in rats [27] and could be similarly effective in neonates. Whilst the underlying neuroprotective mechanism is yet to be understood, RIPostC may interfere with apoptotic pathways by reducing oxidative stress to indirectly inhibit NF-*κ*B activity [27]. In addition, animal models of myocardial injury have shown attenuation of nitrosative stress following postconditioning [28]. We have previously shown in a neonatal piglet model that RIPostC treatment confers white matter protection [29]. In this study we aimed to investigate whether RIPostC protection was associated with changes in oxidative stress, which are also linked to glial activation/inflammation.

## 2. Materials and Methods

### 2.1. Animal Experiments and Surgical Preparation

All animal experiments were approved by the Ethics Committee of the University College London and carried out by licensed personnel in concordance with the UK Home Office Guidelines [Animals (Scientific Procedures) Act, 1986] and in compliance with the ARRIVE guidelines. Large white newborn female piglets at postnatal day 1 (P1) were sedated, anesthetized, and surgically prepared as described previously [30–33]. In brief, animals were sedated with intramuscular midazolam (0.2 mg/kg) and anaesthetised with isoflurane (4% v/v) to facilitate tracheostomy and intubation and maintained with 3% during surgery, 2% v/v otherwise. Mechanical ventilation was titrated to ensure partial arterial pressure of oxygen (PaO_2_) at 8–13 kPa and carbon dioxide (PaCO_2_) at 4.5–6.5 kPa. After the airway was secured, both common carotid arteries were isolated at the level of the fourth cervical vertebra and encircled by remotely controlled vascular occluders (OC2A, In Vivo Metric). An umbilical arterial catheter was inserted to enable continuous monitoring of heart rate (HR), mean arterial blood pressure measurement, and arterial blood extraction to measure PaO_2_, PaCO_2_, pH, electrolytes, glucose (3–10 mmol/L), and lactate (I-Stat, Abbott Laboratories, Maidenhead, UK). Mean arterial blood pressure was maintained at approximately 40 mmHg using saline boluses and infusions of inotropes (dopamine and dobutamine 5–20 *μ*g/kg/min each). Hyperglycemia (>10 mmol/L) was treated by substituting 10% with 5% dextrose; Hyperglycemia (>20 mmol/L) was treated by substituting 5% dextrose for saline. Hyperkalemia (K^+^ > 7 mmol/L) was treated with 4 mcg/kg salbutamol (10 mcg/mL) through the umbilical venous catheter over 10 minutes. Metabolic acidosis (base excess <−10) was corrected with sodium bicarbonate (8.4% wt/vol). All animals received continuous physiological monitoring (SA Instruments, Stony Brook, NY) and extensive life support throughout experimentation. Arterial lines were maintained by infusing 0.9% saline solution (1 mL/h); heparin sodium (1 IU/mL) was added to prevent blockage.

After surgery, and before securing the piglets in a prone position in an acrylic pod with their heads immobilized, the purpose built postconditioning device was placed over both inguinal canals and strapped securely diagonally across the inguinal region with an inflatable bladder placed underneath for further fixation. To assess limb blood perfusion during and after RIPostC, a separate pulse oximeter was secured to the right hind limb, while a laser Doppler assessed perfusion on the left hind limb. An additional pulse oximeter was attached to the right fore limb to measure systemic oxygen saturation [29].

### 2.2. Cerebral Hypoxia-Ischemia

A 70 × 50 mm, elliptical transmit/receive surface coil tuned for ^31^P signal acquisition was secured to the head and the animal placed into the bore of a 9.4 Tesla Agilent spectrometer. Whilst in the MRS system, transient HI was induced by remote occlusion of common carotid arteries using inflatable vascular occluders and reducing fractional inspired oxygen (FiO_2_) to 6% v/v. During HI, the *β*-nucleotide trisphosphate (NTP) was continuously monitored using in-house Matlab (Mathworks) software. When *β*-NTP fell to 40% baseline value, FiO_2_ was titrated to maintain 30–40% *β*-NTP for 12.5 minutes. At the end of insult, the occluders were deflated and FiO_2_ normalized. This leads to loss of neurons and TUNEL and caspase 3 positive cell death as well as microglial activation [31–33].

### 2.3. Experimental Groups

Following resuscitation, piglets were randomised into two groups: HI (*n* = 8) and HI + RIPostC (*n* = 8). Resuscitation after the end of transient HI was taken at time point zero. Piglets were maintained at their target rectal temperature of 38.5 ± 0.5°C using a warmed water mattress. RIPostC was administered with a purpose built device fitted after surgery around the piglet hind limbs and was induced by inflating the device to occlude the femoral artery and then deflating it induced reperfusion. Immediately after resuscitation, piglets underwent 4 cycles of 10 minutes ischemia followed by 10 minutes reperfusion in both hind limbs [29]. In the RIPostC study, cerebral HI resulted in similar insult severity between both groups; however, white matter lactate/N acetyl aspartate was significantly reduced in RIPostC-treated animals 48 hours after HI, and NTP/epp was substantially higher in the treated group [29].

### 2.4. Brain Histology

Forty-eight hours after HI, piglets were euthanized with pentobarbital. The brains were fixed via cardiac perfusion with phosphate-buffered saline (PBS) followed by cold 4% paraformaldehyde/PBS, dissected out and postfixed at 4°C in 2% paraformaldehyde for 9 days. Coronal slices (5 mm thickness) of the whole right hemisphere were embedded in paraffin wax and sectioned to 8 *μ*m thickness. For each animal, 2 sections (bregma 00 and −2.0) were stained issuing immunohistochemistry and 12 different brain regions were blindly examined. To assess nitrosative stress, adjacent brain sections were stained for nitrotyrosine, inducible NO synthase (iNOS), neuronal NO synthase (nNOS), and endothelial NO synthase (eNOS), respectively. To assess glial activation, sections were also stained for glial fibrillary acidic protein (GFAP) and ionized calcium-binding adaptor molecule 1 (IBA1).

The brain sections were treated as previously described [32]. In brief, sections were rehydrated followed by heat treatment for antigen retrieval. Sections were then blocked with 5% goat serum (Sigma-Aldrich, USA) for 30 minutes and incubated overnight with nitrotyrosine (1 : 4000, BD Biosciences, UK), iNOS (1 : 200, Novus Biologicals, UK), nNOS (1 : 200, Bioss, UK), eNOS (1 : 1000, BD Biosciences, UK), IBA1 (1 : 2000 Wako, Japan), GFAP (1 : 1000 EMD Millipore, USA), or GFAP (1 : 5000, eBiosciences, USA). Sections were incubated with biotinylated secondary antibody (1 : 250). The staining was visualized using ABC (Vector Laboratories, UK) and DAB (Thermo Scientific, USA). The sections were counterstained with haematoxylin before being dehydrated in graded alcohol and xylene and mounted with Depex (Leica Biosystems, USA). Naïve controls were stained for the aforementioned markers to demonstrate baseline levels.

For fluorescence double labelling, sections were stained as described above. Nitrotyrosine colabel with eNOS and GFAP sections were incubated with both primary antibodies overnight and then incubated with a biotin-conjugated donkey anti-rabbit Ig and an AlexaFluor488-conjugated goat anti-mouse Ig antibody, followed by tertiary AlexaFluor488-conjugated donkey anti-goat Ig antibody, and Texas Red-Avidin (Vector Laboratories, USA, 1 : 1000) was added. For nitrotyrosine with iNOS, nNOS, and IBA1, sections were incubated with primary, secondary, and tertiary antibodies for nitrotyrosine only, blocked with goat and donkey serum (Sigma-Aldrich, USA) and then incubated with antibodies for the second marker. In both protocols, sections were counterstained with DAPI for nuclear visualisation following incubation with the tertiary antibody and then stored in the dark at 4°C.

### 2.5. Data Analysis

For each animal and section, 12 brain regions were assessed blindly (Figure 2). For nitrotyrosine, nNOS, iNOS, and eNOS, 3 fields per region were scored using a light microscope at ×20 magnification. The semiquantitative scoring system used is described in Table 1 (adapted from [34, 35]). For GFAP and IBA1 assessment was carried out using quantitative thresholding image analysis (adapted from Rahim et al., 2012 [36]). 10 nonoverlapping images (per region) were captured using a live video camera (Nikon, DS-Fi1) mounted onto a Nikon Eclipse E600 microscope at ×40 magnification. Immunoreactivity was determined using Image-Pro Plus (Media Cybernetics, Silver Spring, MD, USA) with the threshold setting kept constant for all subsequent images for each respective antibody.

### 2.6. Statistical Analysis

#### 2.6.1. Comparison of Treatment Groups

For each study and for each measurement type, three separate field (nitrotyrosine, iNOS, eNOS, and NOS) and ten separate field (GFAP and IBA1) results were averaged for each subject for each region. Analysis of variance (ANOVA) was performed on the mean data (overall brain effect) with terms for Treatment, Region, and the interaction between Treatment and Region fitted in the model. The significance (*p* value) for the Treatment effect was assessed to see whether there was evidence of a difference between the overall treatment group means.

#### 2.6.2. Correlation between Measurements

For both treatment groups, the correlation between the different measurements was assessed. A matrix plot which shows a grid of *XY* scatter plots for each possible pairing of two of the measurements was produced with each of the points on the plot being the average values of *X* and *Y* for a particular subject and region. The points in the plot have been identified by the treatment group for the subject and linear regression lines have been plotted so as to assess the strength of a linear relationship between the two measurements. Correlation coefficients *R* have been tabulated for each pairing of variables for each study.

Results are presented as mean (±SD), and statistical significance was assumed for *p* < 0.05.

## 3. Results

### 3.1. Formation of Nitrotyrosine Deposits

**Figure 1:**
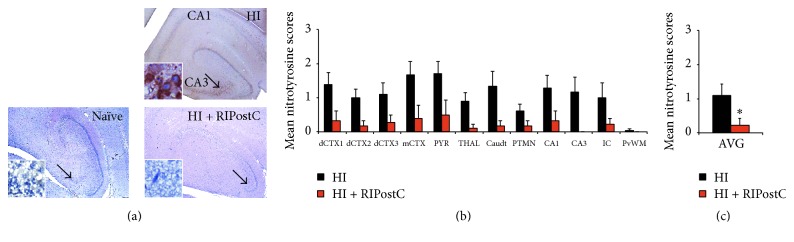
RIPostC decreases nitrotyrosine deposits in the brain 48 hours after HI. (a) Representative nitrotyrosine staining from the hippocampus at 48 hours after HI in HI alone (upper panel) and HI + RIPostC (lower panel). Naïve brain has also been stained to demonstrate the lack of nitrotyrosine deposits in health tissue (left panel). (b) Mean nitrotyrosine scores across multiple brain regions in HI alone and in RIPostC-treated piglets. (c) Overall brain nitrotyrosine scores 48 hours after HI. Data are expressed as mean ± SEM in ANOVA, *n* = 8 per group, and ^*∗*^ *p* < 0.05. dCTX1–3 = dorsal cortex 1–3; mCTX = midtemporal cortex; PYR = pyriform cortex; THAL = thalamus; Caudt = caudate; PTMN = putamen; IC = internal capsule; PvWM = periventricular white matter.

Nitrotyrosine is a reaction product of peroxynitrite, a powerful oxidising and nitrating agent that damages lipids, proteins, and DNA [19, 37]. In human neonates, nitrotyrosine deposits are present after perinatal asphyxia [23] and positive staining of the neuronal cytoplasm is a marker of cell damage, oxidative stress, and inflammation that correlates with severity of brain damage and poor outcomes [38]. However, little to no nitrotyrosine deposits are found in healthy organs [39] or in an infant human case control of spinal muscular atrophy without HIE [23]. In the current study, 48 hours after HI insult, nitrotyrosine staining was seen in all brain regions in both HI (controls) and HI + RIPostC groups. Overall semiquantitative scoring assessment showed that levels of nitrotyrosine deposits 48 hours after HI was significantly reduced across the entire brain hemisphere in RIPostC-treated animals compared to controls (from 1.1 ± 0.3 to 0.2 ± 0.2, *p* = 0.02) (Figure 1(c)).

### 3.2. Production of Oxidative Stress Biomarkers (NO Synthases)

The presence of nitrotyrosine is regarded as a marker of peroxynitrite and suggestive of a role of NO toxicity (nitrosative stress) in HI brain injury. To determine the potential source of nitrotyrosine, NOS –nNOS, iNOS, and eNOS were subsequently assessed using the same semiquantitative scoring system performed in the nitrotyrosine assessment.

#### 3.2.1. Neuronal Nitric Oxide Synthase Expression (nNOS)

**Figure 2:**
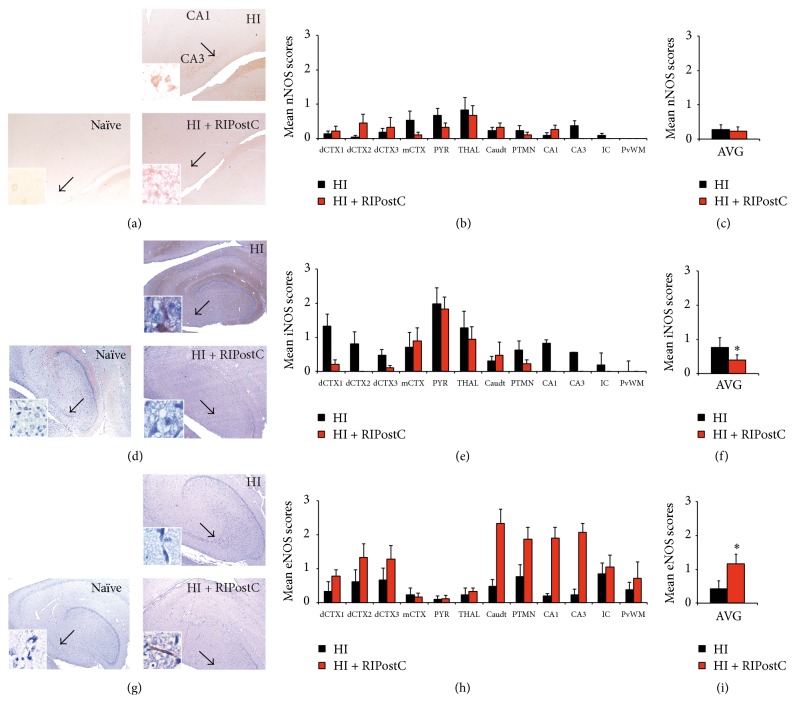
Effect of RIPostC treatment on nitric oxide synthases. (a–c) nNOS levels remain unaffected by RIPostC treatment 48 hours after HI. (a) Representative nNOS staining from the hippocampus at 48 hours after HI in HI alone (upper panel) and HI + RIPostC (lower panel). (b) Mean nNOS scores across multiple brain regions in HI alone and in RIPostC-treated piglets. (c) Overall brain nNOS scores 48 hours after HI. (d–f) RIPostC treatment significantly reduced overall iNOS levels in the brain 48 hours after HI. (d) Representative micrographs of iNOS staining from the hippocampus at 48 hours after HI in HI alone (upper panel) and HI + RIPostC (lower panel). (e) Mean iNOS scores across multiple brain regions in HI alone and in RIPostC-treated piglets. (f) Overall brain iNOS scores 48 hours after HI showing significant reduction in iNOS semiquantitative scoring for the RIPostC-treated group (*p* = 0.010). (g–i) RIPostC treatment was associated with a significant increase in endothelium-derived NO 48 hours after HI injury. (g) Representative eNOS immunohistochemistry from the hippocampus at 48 hours after HI in HI alone (upper panel) and HI + RIPostC (lower panel). (h) Mean eNOS scores across multiple brain regions in HI alone and in RIPostC-treated piglets. (i) Overall significant increase in brain eNOS semiquantitative scores in RIPostC-treated piglets 48 hours after HI. (a, d, g) For comparison, naïve tissue is shown in the left column. Data are expressed as mean ± SEM in ANOVA, *n* = 8 per group, and ^*∗*^ *p* < 0.05. dCTX1–3 = dorsal cortex 1–3; mCTX = midtemporal cortex; PYR = pyriform cortex; THAL = thalamus; Caudt = caudate; PTMN = putamen; IC = internal capsule; PvWM = periventricular white matter.

Assessment of nNOS expression showed no significant difference in nNOS expression in the HI + RIPostC group versus HI alone (Figure 2(c)).

#### 3.2.2. Inducible Nitric Oxide Synthase Expression (iNOS)

We observed significantly less iNOS expression across the brains of the HI + RIPostC group than in the HI control group (from 0.8 ± 0.3 to 0.4 ± 0.2, *p* = 0.010) (Figure 2(f)).

#### 3.2.3. Endothelial Nitric Oxide Synthase Expression (eNOS)

There was a significant overall brain increase in eNOS expression in the HI + RIPostC group compared to the HI control group (from 0.4 ± 0.2 to 1.2 ± 0.3, *p* < 0.0001) (Figure 2(i)).

### 3.3. Glial Activation

To determine whether neuroprotection was associated with glial activation, sections were stained for markers of astrogliosis (GFAP) and microglial activation (IBA1). Analysis of these stainings consisted of quantitative threshold analysis of 10 nonoverlapping images per assessed brain region and immunoreactivity determined using Image-Pro Plus software.

#### 3.3.1. Microglia

**Figure 3:**
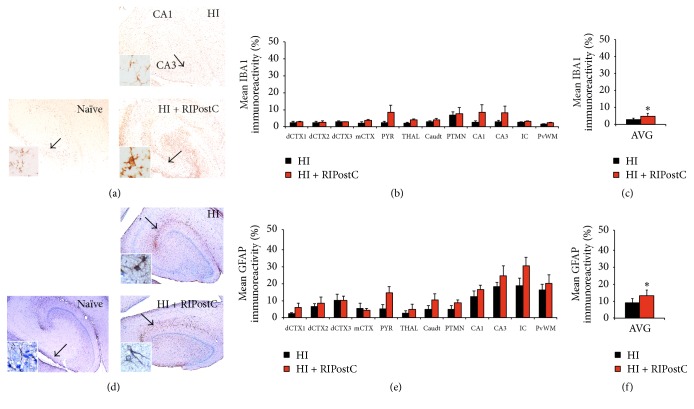
RIPostC-treated animals have increased glial activation. (a–c) IBA1 quantitative thresholding analysis revealed significant increase in microglial IBA1+ staining in the HI + RIPostC group 48 hours after HI. (a) Representative IBA1 staining from the hippocampus at 48 hours after HI in HI alone (upper panel) and HI + RIPostC (lower panel). (b) Mean IBA1 quantitative thresholding analysis across multiple brain regions in HI alone and in RIPostC-treated piglets. (c) Overall IBA1 immunoreactivity was significantly increased in the RIPostC-treated group when compared to HI alone piglets (*p* = 0.006). (d–f) RIPostC treatment significantly increased GFAP+ astrocytes 48 hours after HI. (d) Representative micrographs of GFAP immunohistochemistry from the hippocampus at 48 hours after HI in HI alone (upper panel) and HI + RIPostC (lower panel). (e) Mean GFAP quantitative thresholding analysis across multiple brain regions in HI alone and in RIPostC-treated piglets. (f) RIPostC treatment resulted in an overall significant increase in GFAP+ astrocyte immunoreactivity 48 hours after HI (*p* = 0.002). (a, d) Naïve comparative controls are shown on the left panel. Data are expressed as mean ± SEM in ANOVA, *n* = 8 per group, and ^*∗*^ *p* < 0.05. dCTX1–3 = dorsal cortex 1–3; mCTX = midtemporal cortex; PYR = pyriform cortex; THAL = thalamus; Caudt = caudate; PTMN = putamen; IC = internal capsule; PvWM = periventricular white matter.

The overall level of microglial activation was significantly higher in RIPostC-treated animals in comparison to controls (from 2.8 ± 0.8 to 4.9 ± 1.7, *p* = 0.006) (Figure 3(c)).

#### 3.3.2. Astrocytes

Assessment of astrocyte activation (GFAP) showed overall significant increase in the RIPostC group versus control (from 9.0 ± 2.6 to 13.3 ± 3.4, *p* = 0.002) (Figure 3(f)).

### 3.4. Correlation of Nitrotyrosine, Oxidative Stress Biomarkers, and Glial Activation

Our results showed that nitrotyrosine deposits, expression of NO synthases microglia, and astroglial activation were affected by RIPostC treatment. Therefore, we assessed potential histological correlations between nitrotyrosine deposits and each of the NOS and glial markers used. Overall, nitrotyrosine deposits correlated positively with nNOS (*r* = 0.31) and iNOS (*r* = 0.46) expression and correlated negatively with eNOS expression (*r* = −0.30). Although moderate *R* ^2^ values indicate that these correlations are not very strong, the consistent effect suggests a reasonable link. There was no correlation between nitrotyrosine and IBA-1 (*r* = −0.12) and GFAP (*r* = −0.18) markers (Table 2).

### 3.5. Colocalization of Nitrotyrosine and NOS in Different Cell Types

**Figure 4:**
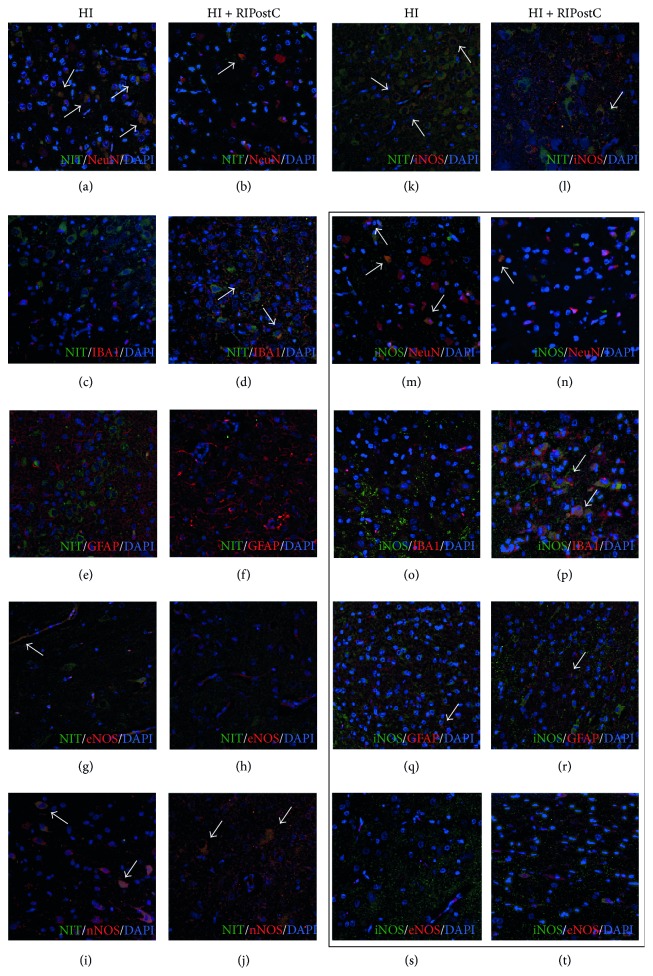
Colocalization of nitrotyrosine deposits and oxidative stress/neural markers. Nitrotyrosine deposits were mostly visible in neurons of HI animals (white arrows, (a)), with some colocalization also present in RIPostC-treated piglets (b). This colocalization was also observed to a much smaller extent in microglia of RIPostC animals (c-d), endothelial cells in the HI group (g-h), and toxic nNOS (i-j) and iNOS (k-l) for both treatments but not in astroglial (e-f). iNOS double labelling (black box) showed its expression in neurons (m-n) and astrocytes (q-r) of both HI and RIPostC animals but not in endothelial cells (s-t). iNOS was also expressed by microglia in the RIPostC group (o-p).

Colabeling of nitrotyrosine with different cell types as well as NOS showed that nitrotyrosine deposits were present mostly in neuronal cells of both treatment groups but to a greater extent in HI animals (Figures 4(a)-4(b)). Nitrotyrosine deposits were also identified in some endothelial cells of HI control animals (Figure 4(g)) as well as nNOS (Figures 4(i)-4(j)) and iNOS positive cells in both groups (Figures 4(k)-4(l)). Some nitrotyrosine deposits were also present in microglia IBA1-stained cells of RIPostC-treated animals (Figures 4(c)-4(d)) but not in astroglia (Figures 4(e)-4(f)). iNOS marker was identified in neurons (Figures 4(m)-4(n)) and astrocytes (Figures 4(q)-4(r)) of both HI and RIPostC-treated animals, as well as in microglia of RIPostC group (Figure 4(p)). However, no iNOS deposits were observed in endothelial cells (Figures 4(s)-4(t)).

## 4. Discussion

Our results demonstrate that RIPostC treatment has a significant association with decrease in nitrotyrosine deposits in the brain, as well as iNOS. Conversely, eNOS was significantly upregulated, with also a substantial increase in IBA1 and GFAP glial markers, suggesting that RIPostC may work through reduction in oxidative/nitrosative stress despite increased glial activation.

### 4.1. Remote Ischemic Postconditioning Reduces Nitrotyrosine Deposits

Several neurological conditions are associated with formation of nitrotyrosine deposits, which in turn is associated with brain tissue damage. Groenendaal et al. described the presence of nitrotyrosine deposits, particularly in the thalamus and inferior olives of 22 full-term infants [23], as well as in the spinal cord of 5 out of 18 full-term neonates [40] who died following HIE. Nitrotyrosine forms from peroxynitrite, a powerful nitrating and oxidising agent that is the reaction product of NO and superoxide anions. Pathological conditions, such as HI, excessively activate neuronal and inducible NOS, leading to elevated NO and subsequent increased peroxynitrite production [19, 41]. This is known to cause oxidative damage to cellular constituents and trigger cell death [42]. Our results demonstrate that RIPostC administered immediately after the end of transient global HI and consisting of 4 cycles of 10-minute hind limb ischemia followed by 4 cycles of 10-minute reperfusion significantly reduced the presence of cytoplasmic nitrotyrosine deposits in the brain 48 hours after HI. The reduction in nitrotyrosine deposits seen with RIPostC treatment indicates a reduction in oxidative/nitrosative stress. This is well known to cause oxidative damage to cellular constituents and trigger cell death [42]. This suggests that one of the protective effects of RIPostC is most likely due to a reduction in upstream production of NO.

### 4.2. Remote Ischemic Postconditioning Reduces iNOS Expression

At the onset of ischemia, nNOS is responsible for the overproduction of NO and consequent cytotoxicity described above [8]. iNOS, which is not normally present in healthy tissue, is activated by a number of inflammatory and immunologic signals [43–45] as well as cerebral ischemia. In contrast to nNOS, iNOS activity peaks in the later stages of ischemic damage [46], suggesting that the NO it generates contributes to delayed cell damage. In neonatal encephalopathy it is the delayed cell death as a result of secondary energy failure that is correlated with the severity of adverse outcomes [47, 48], and iNOS inhibitors have shown protective effects even when administered 24 hours after MCA [49]. Whilst in the current study, nNOS levels were unchanged in the RIPostC group compared with untreated animals; levels of iNOS were significantly reduced. In rats, selective inhibition of iNOS activity with aminoguanidine attenuates postischemic iNOS activity and reduces infarct volume following middle cerebral artery occlusion (MCA) [46]. Similarly, iNOS-knockout mice do not exhibit ischemia-induced iNOS expression and have smaller tissue infarction than wild-type mice after MCA occlusion [50]. Additionally, a study by Wei and colleagues has shown that ischemic postconditioning attenuated iNOS and nitrotyrosine production following focal MCA occlusion [51]. Patients with acute myocardial infarct who underwent ischemic postconditioning demonstrated reduction in iNOS activity in white blood cells as well as decreased plasma nitrotyrosine. The same group showed similar effect in the rat model of myocardial ischemia/reperfusion injury [28]. This suggests that RIPostC may confer neuroprotection in our model by reducing iNOS expression and subsequent production of superoxide to generate peroxynitrite resulting in nitrotyrosine formation.

### 4.3. Remote Ischemic Postconditioning Increases eNOS Expression

In contrast to iNOS and nNOS, eNOS-derived NO has a functionally protective role. In the brain, eNOS is expressed in cerebral endothelial cells [52] and acts as a potent vasodilator; the NO it generates is critical for regulating vascular tone. This facilitates cerebrovascular perfusion and protects against ischemic brain damage by improving blood flow to ischemic tissue [41]. Accordingly, eNOS-knockout mice have reduced cerebral blood flow and enlarged cerebral infarcts after stroke [53]. Conversely, upregulation of eNOS reduces infarct volume after focal [54] and global [55] cerebral ischemia in rats. In the current study, RIPostC-treated animals demonstrated significant upregulation of eNOS expression 48 hours after HI. This is in agreement with the effect of RIPostC on eNOS expression already reported by Peng et al., 2012, where the neuroprotective effect of RIPostC resulted in significant upregulation of eNOS via the PI3K/Akt pathway. This protective effect was reversed following the administration of NG-nitro-L-arginine methyl ester (L-NAME), a NOS inhibitor [17, 55]. Therefore, an increase in eNOS-derived NO probably mediates some of the neuroprotective effect of RIPostC demonstrated in our piglet model of global HI.

### 4.4. Remote Ischemic Postconditioning Activates Microglia

In the current study, levels of IBA1 were significantly increased in RIPostC-treated animals compared to untreated controls. Depending on the extent of injury microglia are classified as either classical (M1) or alternative (M2) and exert neurotoxic or neuroprotective functions, respectively. In ischemic conditions, M1 microglia produce proinflammatory cytokines, reactive oxygen species, and neurotoxic factors and destructively phagocytose tissue whereas M2 produce anti-inflammatory cytokines and neurotrophic factors and phagocytose dying neurons [56, 57]. Accordingly, selective ablation of microglia in mice increases infarct size and apoptosis after stroke [58], but use of microglial inhibitors provides significant neuroprotection against global ischemia [59]. This highlights the complexity of the role of microglia-mediated inflammation in neurological damage or disease. In the current study we observed a significant increase in IBA1 immunoreactivity. This is not in concordance with our previous results, where IBA1 was significantly reduced in the corpus callosum of RIPostC-treated piglets [29]. This difference in results may be a consequence of different analyses, with threshold imaging proving to be a more thorough assessment encompassing a greater area of the different assessed brain regions. The current results raise questions on whether RIPostC treatment is associated with increase in M2 microglial activation or whether there is a neuroprotective effect provided by RIPostC despite microglia activation.

### 4.5. Remote Ischemic Postconditioning Upregulates Astroglial Immunoreactivity

GFAP expression was significantly increased in the RIPostC-treated group. Similarly to NO, there is evidence of a dual role of astrocyte activation following HI. Many studies have shown that attenuation of astrogliosis often correlates with reduced infarct size—nonspecific inhibition of cell proliferation [60] or inhibition of astrocyte component synthesis [61] reduce infarct size accompanied by an attenuated astroglial response. Activated astrocytes secrete a number of proinflammatory cytokines and chemokines, responsible for inducing apoptotic cell death, increasing production of toxic NO, and attracting inflammatory cells to the injured site [62]. However, astroglia also support neurons through providing an antioxidant effect, reducing excitotoxicity and downstream oxidative stress by taking up excess glutamate, and producing neurotrophic factors [63] to reduce injury and promote recovery. Administration of astrocyte-derived factors reduces cerebral oedema and lesion size in rats with induced focal ischemia [64]. GFAP-knockout mice exhibit larger lesions and a greater reduction in cerebral blood flow following focal cerebral ischemia [65]. This suggests that the increase in astrocyte activation seen in the current study with RIPostC treatment could be a contributor to its neuroprotective effect by assisting with vasogenesis. Interestingly, one of the mechanisms in which RIPostC is thought to be involved is the humoral pathway, where circulation of blood-borne protective factors is released by the ischemic limb as well as efferent nerve activation [29]. Therefore, it could be possible that astrocytes may assist with increased blood flow and activation of prosurvival factors and repair mechanisms. The current findings on both microglia and astroglial activation following RIPostC treatment are intriguing and represent a very interesting avenue for further investigation.

## 5. Conclusion

In the piglet model of perinatal asphyxia, RIPostC treatment provided significant white matter protection as observed through TUNEL assay [29]. In the current study, this RIPostC-induced neuroprotection appears to be associated with observed reduced nitrotyrosine deposits across the brain 48 hours after HI. This reduction may be a result of an overall increase in eNOS, as well as reduction of iNOS expression across all assessed brain regions. This RIPostC-mediated reduction in nitrosative stress has also been observed in other in vivo and clinical studies [28, 51, 55]. Interestingly, we have also shown significant increase in astroglial and microglia activation not previously observed. This raises question on whether increased glial activation is somehow protective or whether RIPostC has a beneficial effect reducing nitrosative stress irrespective of glial activation. Furthermore, nitrotyrosine deposits were mostly present in neurons but were also found to a smaller extent in glial cells. Our findings demonstrate the need for better understanding the potential dual role of glial activation following HI. Overall, this study suggests that the neuroprotective effects previously reported are mediated, at least in part, by alteration of nitrosative stress despite glial activation. The effective but safe and noninvasive nature of RIPostC makes it an attractive potential treatment for NE.

## Figures and Tables

**Table 1:** Brain semiquantitative score system.

Score	Staining	% positive cells
0	None	0
1	Weak	≤25
2	Moderate	≥25–≤75
3	Severe	≥75

**Table 2:** Correlation matrix between nitrotyrosine deposits and markers of NOS and glia activity.

RIPostC	eNOS	GFAP	IBA1	iNOS	Nitrotyrosine	nNOS
eNOS		0.22	0.06	−0.29	−0.30	−0.16
GFAP			0.05	−0.20	−0.18	−0.07
IBA1				0.01	−0.12	−0.05
iNOS					0.46	0.36
Nitrotyrosine						0.31
